# Disruptive technologies in mitral modelling—a riot of innovation

**DOI:** 10.1093/icvts/ivac015

**Published:** 2022-02-07

**Authors:** Apurva H Bharucha, Mehdi Eskandari, Olaf Wendler, Max Baghai

**Affiliations:** The Cardiac Care Group, King’s College Hospital, London, UK

**Keywords:** Papillary muscle rupture, Mitral regurgitation, *Ex vivo* model

The anatomical and functional complexities of mitral valve pathologies render clinical decision-making and intervention challenging particularly in emergent settings such as acute papillary muscle (PM) rupture. Although rare since the advent of percutaneous coronary intervention, PM rupture carries a dismal prognosis, with a recent multicentre study published in this journal revealing an in-hospital mortality of 24.9% in those undergoing surgical intervention [1]. Given the rarity of PM rupture, the literature comprises of small retrospective studies producing a paucity of evidence to comprehensively guide patient selection, timing of intervention and the use of bridging strategies Moreover, the limited evidence base precludes a comprehensive understanding of the anatomical, haemodynamic and physiological mechanisms underpinning outcomes in such patients. In this context, Marin-Cuartas *et al.* [2] have developed the an *ex vivo* platform comprising of porcine valves mounted within a left heart simulator to model the haemodynamic effects of acute PM rupture simulated by incrementally cutting chordae to a given PM head. *Ex vivo* mitral models are fundamental to mitral research and this diligently designed study provides an apt reminder of the strengths of this modelling modality in enhancing our understanding of mitral disease processes and spawning research hypotheses especially in areas where the evidence base is limited such as PM rupture. This commendable hypothesis generating work suggests the presence of sub-phenotypic groups within those with PM rupture, the characterization of which could allow for more precise and efficacious interventional strategies and sets the stage for further study in this challenging research area. Given the acuity and rarity of PM rupture, such information would be difficult to derive from clinical studies.

Despite the robust study design, the limitations of *ex vivo* mitral modelling are also apparent. Indeed, porcine models are not patient specific and do not fully emulate the complex biomechanics and anatomical variations of human mitral tissue nor would they simulate the various aetiologies of PM rupture and the concomitant sequalae such as left ventricular dysfunction and arrhythmia. Validation of such models is difficult, thereby limiting their translational value to only experimental use. Large-scale use of animal tissue within *ex vivo* simulators may also be costly and carry ethical implications.

The advent and rapid evolution of disruptive technologies in medicine—namely three-dimensional (3D) printing, computational modelling, machine learning and extended realities—and advances in cardiac imaging have the scope to revolutionize mitral modelling. Moreover, they bear the translational potential to facilitate ‘personalized’ cardiac care and improve clinical outcomes by offering patient-specific procedure planning, surgical simulation training and procedural augmentation. Such technologies are also capable of being integrated with *ex vivo* platforms, therefore enhancing their effectiveness. Indeed, it is unlikely that *ex vivo* platforms will be rendered obsolete with the advent of these novel technologies.

By integrating volumetric cardiac imaging, material technologies and software engineering, 3D printing transforms digital objects into 3D replicas through multi-layered material deposition over a digitally defined geometry. Advances in cardiac imaging—notably in cardiac CT and 3D transoesophageal echo (TOE)—enable rendering of highly accurate 3D images of cardiac structures. Progress in software engineering, particularly with the integration of machine learning (ML), now enables highly accurate and rapid delineation of anatomical boundaries in a process known as segmentation [3]. Meanwhile, polyjet printers could enable the fusion of multiple printing materials to better emulate the structural complexities of human valves. Therefore, having begun from simply 3D printing mitral valve replicas for anatomical observation of pathology, it is now feasible to create cost-effective patient-specific deformable mitral valve replicas, which can be modelled within a left heart simulator using TOE, thereby enabling haemodynamic and imaging characterization of mitral pathologies as demonstrated by Ginty *et al.* [4]. Such platforms carry the potential for incorporation into patient-specific procedure planning, the evaluation of novel mitral technologies and simulation training.

The domain agnostic nature of ML enables identification of complex associations within big data with the efficacy of such models increasing with volume of inputted data. The quantitative nature of structural heart imaging naturally lends itself to ML and such methods have found a myriad of applications in mitral modelling particularly in automating and enhancing mitral segmentation to rapidly produce accurate 3D mitral replicas in the quest for point of care 3D printing. ML methods and 3D printing have also been successfully integrated to produce a hyper-realistic simulation platform for training in minimally invasive mitral valve surgery [5].

Extended realities technologies harbour promise in the arena of mitral valve modelling and intervention particularly in surgical training, virtual proctoring and procedure planning [6]. There is also the tantalizing prospect of intraprocedural integration as demonstrated by Chu *et al.* where augmented reality optimized intraprocedural TOE guidance facilitated more safer NeoChord implantation in a porcine model [7].

Despite the rapid evolution of disruptive technologies, there is cause for caution amidst this riot of innovation. These technologies are relatively nascent, costly and carry their own inherent limitations. Indeed 3D-printed mitral replicas are as yet unable to accurately model mitral tissue and the complexities of mitral biomechanics such as deformation. The accuracy of 3D-printed replicas is also dependent on the quality of cardiac imaging from which they are derived and despite significant advances in this area, important limitations still apply. Computational flow dynamics offer high fidelity simulation of stress and deformation, but widespread clinical application is stifled by cost and complexity. Meanwhile, extended reality technologies face technical challenges associated with interfacing high volume multimodality data and issues associated with motion induced sickness in operators. Headsets can be cumbersome and could cause procedural hindrance. Much of the research with these technologies comprises of small proof of feasibility studies and comparison between studies is challenging given their heterogeneity.

In essence, disruptive technologies look set to continue their rapid evolution and play an instrumental role in the quest for personalized cardiac care; however, careful consideration is required to their limitations when contemplating widespread clinical translation. Pragmatism would dictate that hybrid modelling through integration of these technologies’ may optimize their efficacy and this may represent the next translational step in this exciting field.
